# Supplementary material for: Functional recruitment of dynamin requires multimeric interactions for efficient endocytosis
Source: Nat Commun. 2019 Oct 1;10:4462. doi: 10.1038/s41467-019-12434-9 (PMC6773865; doi:10.1038/s41467-019-12434-9)
Supplement: Supplementary file 2 — Reporting Summary [file 41467_2019_12434_MOESM2_ESM.pdf]

nature research

Corresponding author(s): *Double-blind peer review submissions: write DBPR and your manuscript number here instead of author names.*

Last updated by author(s): *YYYY-MM-DD*

Reporting Summary

Nature Research wishes to improve the reproducibility of the work that we publish. This form provides structure for consistency and transparency in reporting. For further information on Nature Research policies, see Authors & Referees and the Editorial Policy Checklist.

Statistics

For all statistical analyses, confirm that the following items are present in the figure legend, table legend, main text, or Methods section.

n/a	Confirmed	
☐	☒	The exact sample size (*n*) for each experimental group/condition, given as a discrete number and unit of measurement
☐	☒	A statement on whether measurements were taken from distinct samples or whether the same sample was measured repeatedly
☐	☒	The statistical test(s) used AND whether they are one- or two-sided<br>*Only common tests should be described solely by name; describe more complex techniques in the Methods section.*
☒	☐	A description of all covariates tested
☐	☒	A description of any assumptions or corrections, such as tests of normality and adjustment for multiple comparisons
☐	☒	A full description of the statistical parameters including central tendency (e.g. means) or other basic estimates (e.g. regression coefficient) AND variation (e.g. standard deviation) or associated estimates of uncertainty (e.g. confidence intervals)
☐	☒	For null hypothesis testing, the test statistic (e.g. F, t, r) with confidence intervals, effect sizes, degrees of freedom and P value noted<br>*Give P values as exact values whenever suitable.*
☒	☐	For Bayesian analysis, information on the choice of priors and Markov chain Monte Carlo settings
☒	☐	For hierarchical and complex designs, identification of the appropriate level for tests and full reporting of outcomes
☐	☒	Estimates of effect sizes (e.g. Cohen's d, Pearson's r), indicating how they were calculated

Our web collection on statistics for biologists contains articles on many of the points above.

Software and code

Policy information about availability of computer code

Data collection	Live and fixed cell imaging was collected on setups equipped with Metamorph software (version 7.8, Molecular Devices)<br>Electrophysiology data was recording on a HEKA EPC10 amplifier run by PatchMaster.<br>Surface plasmon resonance data was obtained on Biacore X100 and T200 systems (GE Healthcare Life Sciences)
Data analysis	Live and fixed cell imaging data was analysed with Metamorph 7.8 and custom written Matlab 2018 macros. Statistical testing was performed with GraphPad Prism 8.

For manuscripts utilizing custom algorithms or software that are central to the research but not yet described in published literature, software must be made available to editors/reviewers. We strongly encourage code deposition in a community repository (e.g. GitHub). See the Nature Research guidelines for submitting code & software for further information.

Data

Policy information about availability of data

All manuscripts must include a data availability statement. This statement should provide the following information, where applicable:
- Accession codes, unique identifiers, or web links for publicly available datasets
- A list of figures that have associated raw data
- A description of any restrictions on data availability

Data supporting the findings of this manuscript are available from the corresponding author upon reasonable request. A reporting summary for this Article is available as a Supplementary Information file.

The source data underlying Figs. 1e,f, 2b-d,g, 4b,e, 5d and Supplementary Figs 1, 2 and 6 are provided as a Source Data file.

Field-specific reporting

Please select the one below that is the best fit for your research. If you are not sure, read the appropriate sections before making your selection.

☒ Life sciences ☐ Behavioural & social sciences ☐ Ecological, evolutionary & environmental sciences

For a reference copy of the document with all sections, see nature.com/documents/nr-reporting-summary-flat.pdf

Life sciences study design

All studies must disclose on these points even when the disclosure is negative.

Sample size	Sample size for biophysical measures was triplicate experiment on the same protein and peptide production.<br>Cell sample size for imaging was at least five in at least two separate experiments.
Data exclusions	No data was excluded post analysis if criteria for good recordings were met (stability of event frequency, criteria for electrophysiological recordings as described in methods).
Replication	All the results could be replicated except the proteomics analysis which was done on a single sample.
Randomization	All conditions were tested with enough repetitions (fixed and live cells) for statistical tests to be applicable.
Blinding	All experiments involving several conditions for cellular imaging were performed blind, including the analysis and decision to keep the recording for further analysis. Blinding was revealed only after full analysis and quantification of the recording.

Reporting for specific materials, systems and methods

We require information from authors about some types of materials, experimental systems and methods used in many studies. Here, indicate whether each material, system or method listed is relevant to your study. If you are not sure if a list item applies to your research, read the appropriate section before selecting a response.

Materials & experimental systems

n/a	Involved in the study	
☐	☒	Antibodies
☐	☒	Eukaryotic cell lines
☒	☐	Palaeontology
☐	☒	Animals and other organisms
☒	☐	Human research participants
☒	☐	Clinical data

Methods

n/a	Involved in the study	
☒	☐	ChIP-seq
☒	☐	Flow cytometry
☒	☐	MRI-based neuroimaging

Antibodies

Antibodies used	anti-dynamin Santa Cruz sc-6401<br>anti-GFP Sigma 11814460001
Validation	anti-dynamin: No signal in the dynamin TKO cell line (Figure 1B) ;<br>anti-GFP; commercial web site data https://www.sigmaaldrich.com/catalog/product/roche/11814460001?lang=fr&region=FR

Eukaryotic cell lines

Policy information about cell lines

Cell line source(s)	NIH 3T3 cells : ECACC 93061524<br>BSC-1 cells: ECACC 85011422<br>MEF Dynamin TKO cells : gift from the laboratory of Pietro De Camilli (Yale University)<br>SK-MEL-2 cell clone Ti95 (Dnm2-GFPen all edited) was a gift from David Drubin (University of California, Berkeley, CA)
Authentication	No cell line was authenticated beyond the standard cell culture practice (one cell line at a time in the culture hood...)
Mycoplasma contamination	All cell lines tested negative for mycoplasma
Commonly misidentified lines<br>(See ICLAC register)	NA

Animals and other organisms

Policy information about studies involving animals; ARRIVE guidelines recommended for reporting animal research

Laboratory animals	Rat, Sprague Dawley, male, 2-3 months old
Wild animals	NA
Field-collected samples	NA
Ethics oversight	Killing procedure in accordance with the European 2010/63/EU directive for killing animals. Approval by the Bordeaux University Ethics Committee (CE50).

Note that full information on the approval of the study protocol must also be provided in the manuscript.
